# An Interprofessional Training Ward in Pediatric Cardiology: Ensuring Patient Safety and Results from the Evaluation of Patient and Parent Satisfaction

**DOI:** 10.3390/children12111541

**Published:** 2025-11-14

**Authors:** Anthea Peters, Wiebke Spree, Tobias Kratz, Soyhan Bagci, Johannes Breuer

**Affiliations:** 1 Department of Pediatric Cardiology, Children’s Hospital, University of Bonn, 53127 Bonn, Germanyjohannes.breuer@ukbonn.de (J.B.); 2 Pediatric Emergency Center, Children’s Hospital, University of Bonn, 53127 Bonn, Germany; soyhan.bagci@ukbonn.de

**Keywords:** interprofessional training ward, congenital heart defect, safety protocol, pediatric cardiology, interprofessional education, patient safety

## Abstract

Background/Objectives: Interprofessional training wards (ITWs) are effective in fostering interprofessional collaboration during undergraduate medical training. Ensuring safety is particularly crucial for vulnerable patient groups. We developed a safety concept for the pediatric cardiology ITW, enabling nursing trainees and final-year medical students to independently care for children with congenital heart defects (CHDs). This study aims to evaluate whether our safety concept allows the inclusion of patients with CHDs in the care provided by our ITW. It also seeks to evaluate patient feedback, including their perception of safety, and to investigate whether there is a correlation between the input and the severity of the heart defect. Methods: From 2020 to 2023, 16 ITW blocks were evaluated, each lasting 3–4 weeks. The three-stage safety concept includes patient selection, emergency prevention and emergency training. CHD severity in treated patients was recorded, and experiences were assessed via parent/patient questionnaires. Results: Between August 2020 and November 2023, 183 patients with mild (n = 52), moderate (n = 28), and severe (n = 103) CHDs were treated. The severity of CHDs was generally higher compared to other pediatric cardiology wards in Germany. There was no significant difference between the severity of CHDs of the patient treated by the ITW and those treated on the regular ward. Of 140 completed questionnaires, 99% of respondents would recommend the program. Overall impressions ranged from “very good” (81%) to “good” (19%), with a high sense of safety reported. Positive free-text comments highlighted the team’s competence and friendliness. Conclusions: The safety concept enabled the successful treatment of patients across all CHD severities, providing a transferable model for safe, interprofessional care in ITWs.

## 1. Introduction

Challenges in communication and collaboration among healthcare professionals can adversely affect patient care. Effective communication and teamwork are therefore critical for ensuring positive patient outcomes and maintaining patient safety [1,2,3]. To address these challenges, the World Health Organisation (WHO) advocates for interprofessional collaboration as a key strategy to meet the increasing demands of modern healthcare systems. To achieve this goal, interprofessional competencies must be integrated into medical education. The WHO defines interprofessional education as follows: *“Interprofessional education occurs when two or more professionals learn about, from, and with each other to enable effective collaboration and improve health outcomes”* [4].

Interprofessional training wards (ITWs) have been introduced to promote practical, joint, and patient-centred interprofessional education [5]. In ITWs, trainees from various healthcare professions collaborate to provide patient care under the supervision of experienced nurses and physicians [6,7,8]. This structure encourages interprofessional learning by granting trainees a high degree of responsibility and autonomy [9,10]. Furthermore, students experience a high level of authenticity, which enhances learning [11]. Typically, the participating students are in their final year of education, allowing for a high level of self-reliance and independent decision-making [8]. Research has demonstrated that ITWs enable safe patient care by medical students and nursing trainees [12,13,14,15]. Previous studies in pediatric settings indicate that interprofessional training wards are feasible and well accepted by both patients and families, even in children as young as one year old [16]. High levels of satisfaction and perceived safety have been reported, alongside improvements in interprofessional collaboration and communication skills among trainees [17]. Recent evaluations also highlight positive effects on long-term professional development and confidence in interprofessional teamwork, as well as on the quality of patient care [18]. Additional research demonstrates that structured interprofessional learning activities in pediatric contexts can enhance collaboration in emergencies and improve both perceived and observed clinical performance [19]. Despite these encouraging findings, evidence from highly specialised pediatric fields, such as cardiology, remains limited, underscoring the need for further research on the safety, feasibility, and transferability of these models.

Children and adolescents with severe or chronic conditions, such as congenital heart defects (CHD), pose unique challenges. In pediatric cardiology wards, trainees are usually entrusted with fewer independent tasks due to safety concerns, and currently, no data exist on the safety of including such patients in ITWs. To address this gap, we developed a specialised safety concept that enables nursing trainees and medical students to provide independent care for this vulnerable patient group while ensuring patient safety.

Since 2019, our clinic has operated an ITW (Kinder-IPSTA) on the pediatric cardiology ward. Here, nursing trainees and final-year medical students collaboratively care for pediatric cardiology patients. The medical students are responsible for day-to-day ward management, including conducting patient admissions and discharges, ordering diagnostic tests and laboratory investigations, and leading ward rounds for their assigned patients. They perform these tasks autonomously within their defined scope of practice, while a board-certified pediatrician and an experienced nurse act as an ITW supervisor and remain continuously present on the ward. Residents are assigned to other patients and are not involved in the care of IPSTA patients during these rotations. The overall responsibility for patient care lies with the licensed physicians and nursing staff serving as supervisors. As this project was conducted in a tertiary teaching hospital in Germany, the involvement of medical students and nursing trainees in patient care under supervision is standard practice and fully compliant with national medical and nursing education regulations.

Typically, two nursing trainees and two final-year students work in pairs across two shifts. Each shift includes one nursing trainee and one final-year medical student who jointly care for two to three patients, under the supervision of one nurse and one physician. Each patient was reviewed by the supervising physician at least once daily (usually twice) and by the supervising nurse two to three times daily, in addition to the trainees’ ongoing bedside care. The Kinder-IPSTA runs 4–5 times annually in 3–4-week blocks, coordinated with the alternating school-based and practical training schedules of the nursing trainees. Block duration is determined by the overlap between nursing trainees’ and medical students’ rotations. Preparation includes an e-learning module and an introductory day, during which feedback techniques are practiced, team rules are defined, and the ISBAR-R handover model is introduced. The safety concept is emphasised early, with e-learning content covering Crisis Resource Management (CRM) principles and basic pediatric CPR techniques. On the introduction day, practical pediatric basic life support (PBLS) training is conducted using baby and toddler manikins. This is followed by a pediatric advanced life support (PALS) simulation, including a detailed review of the safety concept. During the ward rotation, teams participate in structured reflections several times a week using the Korthagen reflection cycle (similar to Kolb’s) [20]. Weekly training sessions focus on learning strategies, task prioritisation, and patient safety. Midway through each block, participants receive individual feedback, and the program concludes with a final day that includes 360° feedback and reflection on professional roles and learning experiences.

This study aims to demonstrate that nursing trainees and final-year medical students can independently provide comprehensive care for high-risk patients in pediatric cardiology wards. We present the specialised safety concept developed for this purpose and evaluate patient and parent satisfaction with various aspects of care at the Kinder-IPSTA, including their perception of safety and security.

Research questions and hypotheses:

1. What are the results of applying the safety concept in pediatric patients with severe congenital heart defects in relation to their safe admission to the ITW?

**Hypothesis H1.** 
*The safety concept allows patients with severe CHD to be included in the care provided by our ITW*.

**H1_0_.**  *The safety concept does not allow patients with severe CHD to be included in the care provided by our ITW*.

2. How do pediatric cardiology patients and parents rate their experience at the ITW?

**H2.**  *Pediatric cardiology patients and parents evaluate their experience at the ITW primarily positively rather than negatively*.

**H2_0_.**  *Pediatric cardiology patients and parents rate their experience at the ITW primarily negatively rather than positively*.

3. Do patients with severe CHD and parents feel safe during their time at the ITW?

**H3.**  *Patients with severe CHD and parents feel more safe than unsafe during their time at the ITW*.

**H3_0_.**  *Patients with severe CHD and parents feel more unsafe than safe during their time at the ITW*.

4. Is there a correlation between the perceived quality of care at the ITW and the severity of the CHD?

**H4.**  *There is a correlation between the perceived quality of care at the ITW and the severity of CHD*.

**H4_0_.**  *No correlation exists between the perceived quality of care at the ITW and the severity of CHD*.

## 2. Materials and Methods

### 2.1. Safety Concept

A three-stage safety concept has been developed to ensure the safe treatment of pediatric cardiology patients during the ITW. This approach is intended to reduce the likelihood of emergencies and provide high-quality, guideline-based treatment should one occur.

The first stage consists of selecting suitable patients for the Kinder-IPSTA. Patients with a high risk of sudden emergencies need to be excluded. However, the most comprehensive range of congenital heart defects and age groups should be included simultaneously. Based on these preliminary considerations, patients with relevant cardiac arrhythmias and those who had experienced a resuscitation event within the last 24 h were excluded from treatment in the Kinder-IPSTA. In addition, patients with ductus-dependent congenital heart defects were excluded from Kinder-IPSTA treatment. No age restrictions were imposed, so the large proportion of infants on a pediatric cardiology ward could also be included in therapy at the Kinder-IPSTA. The selection of patients for the next day is carried out the day before by the ITW supervisors, together with the ward’s shift manager. On average, 2–5 new patients are admitted to the pediatric cardiology ward each day. After applying the predefined exclusion criteria, the remaining eligible patients are reviewed. From these, 1–2 patients per day (a total of 2–4 patients at a time) are selected for inclusion in the Kinder-IPSTA based on educational and clinical considerations. The level of training and knowledge of the Kinder-IPSTA participants, as well as the expected degree of illness and care required by the patients, are also taken into account when assigning cases. The final decision is made jointly by the supervising physician and nurse after discussing these factors to ensure an appropriate educational and clinical balance. The supervising physicians and nurses aim to ensure a representative mix of age groups, disease severity, and expected care requirements while maintaining patient safety and an appropriate supervision ratio.

**Figure 1:**
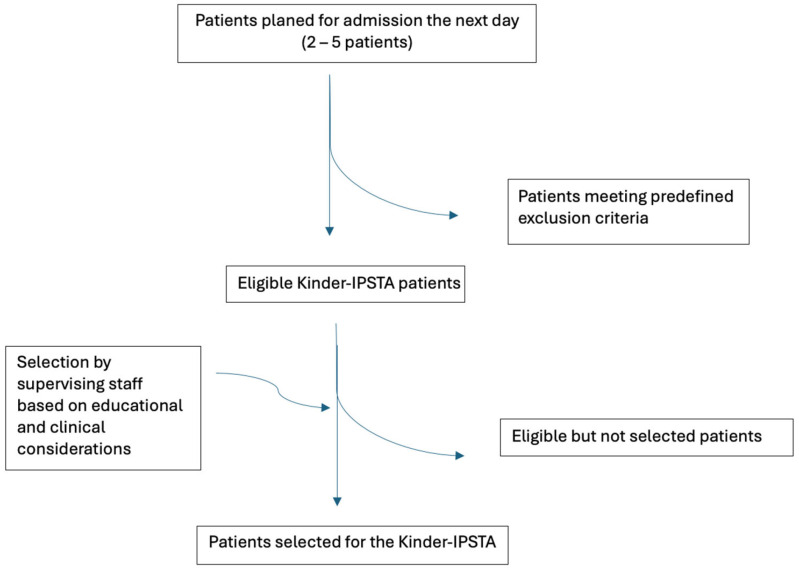
Schematic overview of the patient selection process for the Kinder-IPSTA.

Because this process is integrated into the daily ward workflow and not all eligible but non-included patients are systematically documented, a STROBE-style inclusion flowchart could not be constructed. This pragmatic approach reflects real-world clinical conditions, where prospective logging of all eligible but non-selected patients is not feasible in routine care. Instead, Figure 1 provides a schematic overview of the patient selection process.

The second stage involves the early detection of potential deterioration and critical situations. This enables timely intervention without an emergency arising. To ensure this, patients are assessed at least once per shift by the supervising physician and at every care round by the supervising nurse. These assessments identify unexpected deterioration in the patient’s condition at an early stage, enabling prompt initiation of appropriate measures. During the morning ward round, the participating trainees discuss which parameters require particular attention for each patient and identify potential complications.

If patients admitted to the Kinder-IPSTA project deteriorate unexpectedly in the meantime, participants must inform their ITW supervisors (medical and nursing) without delay. A re-evaluation of the patients is then carried out with the participants, and a decision is made on whether the patients can remain in the Kinder-IPSTA or should be transferred to the ward’s regular care.

Finally, the third stage is about ensuring good, guideline-based care in the event of an unexpected emergency. To ensure the best possible workflow in an emergency, all Kinder-IPSTA participants received a theoretical introduction to the cardiopulmonary resuscitation treatment algorithm for infants and children via e-learning. On the training day, this theoretical knowledge is supplemented by a pediatric basic life support course using blended learning, with two simulation scenarios in which participants need to care for an unstable pediatric patient. In a real resuscitation event, the familiar in-house algorithm (call for help, initiate basic resuscitation, alert the MET team) is followed. The basic resuscitation procedure is coordinated by supervising nurses and physicians, with the support of ward staff and participants, and handed over to the MET team upon arrival.

### 2.2. Patient Questionnaire

**Figure 2:**
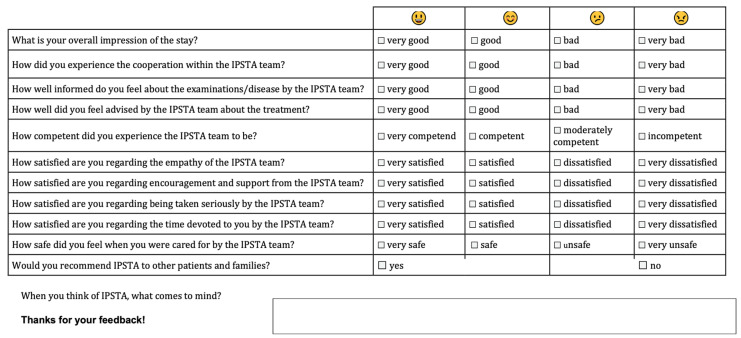
Patient and parent questionnaire on the Kinder-IPSTA.

All parents of patients involved in the Kinder-IPSTA project were provided with an anonymised evaluation form. Once patients were able to contribute independently, they too were invited to participate in the evaluation process. A single questionnaire was used for both parents and children. To ensure that children of various ages could respond meaningfully, the identical items were accompanied by smiley-face icons representing the four-point Likert scale and by simplified wording. This allowed children to complete the form independently or with parental assistance (Figure 2).

**The following questions were evaluated:**

What is your overall impression of the stay?

(1 = very good, 2 = good, 3 = bad, 4 = very bad)

How did you experience the cooperation within the IPSTA team?

(1 = very good, 2 = good, 3 = bad, 4 = very bad)

How well informed do you feel about the examinations/disease by the IPSTA team?

(1 = very good, 2 = good, 3 = bad, 4 = very bad)

How well did you feel the IPSTA team advised you about the treatment?

(1 = very good, 2 = good, 3 = bad, 4 = very bad)

How competent did you experience the IPSTA team to be?

(1 = very competent, 2 = competent, 3 = moderately competent, 4 = incompetent)

How satisfied are you with the IPSTA team’s empathy?

(1 = very satisfied, 2 = satisfied, 3 = dissatisfied, 4 = very dissatisfied)

How satisfied are you with the encouragement and support from the IPSTA team?

(1 = very satisfied, 2 = satisfied, 3 = dissatisfied, 4 = very dissatisfied)

How satisfied are you regarding being taken seriously by the IPSTA team?

(1 = very satisfied, 2 = satisfied, 3 = dissatisfied, 4 = very dissatisfied)

How satisfied are you with the time the IPSTA team has devoted to you?

(1 = very satisfied, 2 = satisfied, 3 = dissatisfied, 4 = very dissatisfied)

How safe did you feel when you were cared for by the IPSTA team?

(1 = very safe, 2 = safe, 3 = unsafe, 4 = very unsafe)

Would you recommend IPSTA to other patients and families?

(1 = yes, 2 = no)

When you think of IPSTA, what comes to mind?

No validated instrument for patient satisfaction specific to pediatric interprofessional training wards currently exists. Therefore, the questionnaire was developed by the study team, reviewed by content experts, and pilot-tested with 10 families to ensure clarity and comprehensibility, acknowledging that the absence of formal validation represents a methodological limitation.

Because the instrument includes distinct content domains, we did not conduct psychometric validation, such as factor analysis or internal consistency testing.

In addition, statistical data were collected on age, gender, length of stay at IPSTA, and total treatment duration in the hospital. The questionnaires were collected in a locked letterbox on the ward, allowing patients and their parents to provide anonymous critical feedback on the project.

### 2.3. Patients

ITW patients from 16 ITW rounds from August 2020 to November 2023 were included. Current diagnoses and medical letters were available for all patients. The majority of patients included in the Kinder-IPSTA were non-surgical cases, most commonly admitted for diagnostic or interventional cardiac catheterisation. In addition, clinically stable children awaiting cardiac surgery or admitted for preoperative optimisation were also cared for on the ward. They could be included if they met all predefined safety criteria.

The ITW patients were categorized into three severity levels—mild, moderate, and severe—based on their congenital heart defects, following the classification system used in the PAN study [21]. Examples of mild defects include small or muscular ventricular septal defects and atrial septal defects. Moderate defects comprise conditions such as atrioventricular septal defects or coarctation of the aorta, while severe defects include complex malformations such as single-ventricle physiology or Pulmonary atresia. This classification was chosen because experts use it to classify congenital heart defects, and it not only refers to the post-operative phase but also provides comparability with other German clinics. The data on the nationwide prevalence and severity of congenital heart defects from July 2006 to June 2007, collected as part of the PAN study, served as an external reference for assessing disease severity in the Kinder-IPSTA cohort.

### 2.4. Statistics

All data analysis was performed in IBM SPSS Statistics 29 software (IBM, New York, NY, USA). The dataset was cleaned by checking for incomplete entries and verifying internal consistency. Missing values occurred when questionnaire items were left unanswered by patients or parents. They were excluded on an item-by-item basis.

Descriptive statistics for the sample were compiled. The patients included in the Kinder-IPSTA were compared with respect to CHD severity, and the findings of the PAN Study on the distribution of severity treated in university hospitals/departments of pediatric cardiology in Germany were used as a reference (Hypothesis 1). A Chi-Square test was conducted on the 100 randomly selected patients and the control group to evaluate whether a significant difference in severity grades existed (Hypothesis 1). The Kruskal–Wallis test and Chi-square test were used to detect statistically significant differences in age, sex, CHD severity, and length of stay across the groups. For the questionnaire responses, Likert-scale responses are represented by the frequency distribution. (Hypotheses 2 and 3). A Mann–Whitney U Test was used to determine whether there were differences in questionnaire item ratings between patient groups with different severities of CHD (Hypothesis 4) and between patient and parent assessments. Pearson correlations were performed to determine relationships among overall impression, safety, and the other questionnaire items. Stepwise multiple linear regression analyses (entry criterion *p* ≤ 0.05; removal criterion *p* ≥ 0.10) were performed to identify independent predictors of overall impression, perceived safety, and recommendation. Regression results are reported with standardized β coefficients, 95% confidence intervals, and model fit indices (R^2^, adjusted R^2^, F, and *p*-values). Statistical significance was set at *p* < 0.05.

### 2.5. Qualitative Evaluation

Free-text responses were categorised as positive or negative and analysed inductively by three members of the project team. Coding was first conducted individually and subsequently discussed within the group until consensus was reached. Discrepancies were resolved through discussion, and agreement was achieved for all comments; therefore, no inter-rater reliability coefficient was calculated. Thematic saturation was assumed when no new categories emerged.

## 3. Results

Sixteen Kinder-IPSTA runs were evaluated from August 2020 to November 2023 on the pediatric cardiology ward at the University Children’s Hospital in Bonn. There were 12 runs lasting 3 weeks and 4 runs lasting 4 weeks. During the study period, 32 nursing trainees and 29 medical students participated in the project.

### 3.1. Descriptive Data

The ITW team treated 183 patients. For 11 patients, no retrospective classification could be made, and for 2 patients the disease data were incomplete. Complete data were available for 170 patients treated by the ITW team. Patient characteristics are presented in Table 1.

Regarding sex, age, length of stay, and severity, the Kruskal–Wallis test showed no significant difference between the 16 runs.

**Figure 3:**
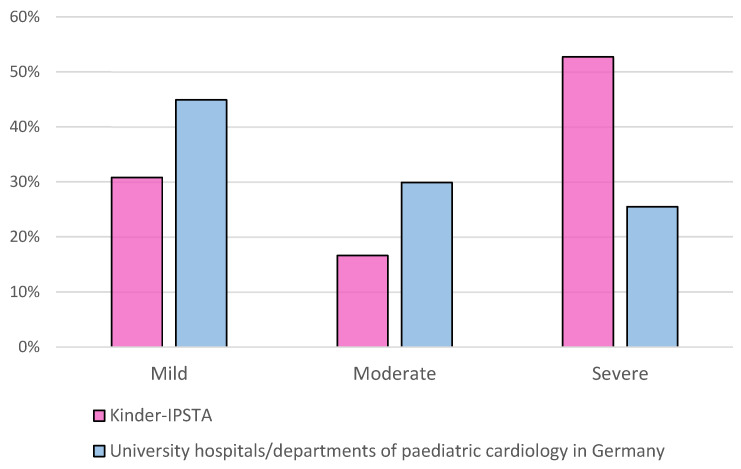
Distribution of severity of congenital heart defects on the Kinder-IPSTA in comparison with University hospitals/departments of pediatric cardiology in Germany, according to the PAN study.

We hypothesised that the safety concept allows patients with severe congenital heart defects to be included in the care provided by our Kinder-IPSTA (H1). Figure 3 illustrates the classification of patients according to the varying degrees of CHD severity (mild/moderate/severe) compared with data from the PAN study for university hospitals/departments of pediatric cardiology in Germany.

### 3.2. Evaluation of the Questionnaires

We hypothesised that pediatric cardiology patients and parents would primarily evaluate their experience at the Kinder-IPSTA positively rather than negatively (H2) and that patients with severe congenital heart defects and parents would feel relatively safer than unsafe during their time at the Kinder-IPSTA (H3).

A total of 149 questionnaires were analysed. Parents of patients completed 103 (72%) questionnaires, patients completed 33 (23%), and both patients and their parents completed 7 (5%). The evaluation of the individual items is shown in Table 2.

In response to the question: “Would you recommend Kinder-IPSTA to other patients and families?”, Ninety-nine per cent of parents and patients (n = 147) answered “Yes” (all but one of the questionnaires completed by parents).

There were no significant differences in the evaluations by parents or patients, as indicated by the Mann–Whitney U Test (*p* > 0.05 for all items). Perceived safety ratings did not differ significantly between parents and patients (*p* = 0.075, *U* = 1883.0, *Z* = −1.78, *r* = 0.15, 95% CI [0.00–0.29]). The patients rated their safety perceptions higher than their parents did (Table 3).

Regarding the questionnaire items, the Mann–Whitney U Test showed no significant difference between the runs (*p* > 0.05).

### 3.3. Regression Analyses

The Pearson test revealed no significant correlations between age, gender, or length of stay and the questionnaire items.

We hypothesised that there would be a correlation between the perceived quality of care at the Kinder-IPSTA and the severity of congenital heart defects (H4).

To test whether the severity of the CHD influences perceived safety in the care provided by the ITW team, a Mann–Whitney U Test was performed.

There were no significant differences among the groups of patients with mild, moderate, and severe congenital heart defects in the questionnaire items, as determined by the Kolmogorov–Smirnov test (*p* > 0.05). In particular, no differences were found concerning perceived safety (*p* = 0.428, H(2) = 0.79, η^2^ = 0.01, 95% CI [0.00–0.06]). The patients and their parents seemed to feel very safe despite severe heart disease. Only in one questionnaire did the parents report feeling unsafe during care (Table 4).

In the stepwise multiple linear regression model with perceived safety as the dependent variable (predictors: empathy, competence, cooperation, information, advice, support, time, taken seriously), the model was significant (*R*^2^ = 0.56, adjusted *R*^2^ = 0.54, *F*(5, 138) = 34.6, *p* < 0.001). Significant positive predictors included competence (β = 0.31, *p* < 0.001), empathy (β = 0.30, *p* < 0.001), advice (β = 0.28, *p* < 0.001), and time devoted (β = 0.29, *p* < 0.001). “Taken seriously” showed a small but significant negative association (β = −0.20, *p* = 0.014), suggesting partial collinearity among communication-related factors. Together, these predictors explained 55.6% of the variance in perceived safety, indicating that competence, empathy, advice, and sufficient time were the strongest contributors to a sense of safety during Kinder-IPSTA care.

Pearson correlations indicated that overall impression was strongly associated with empathy (*r* = 0.61, *p* < 0.001, 95% CI [0.48–0.71]), cooperation (*r* = 0.54, *p* < 0.001, 95% CI [0.40–0.65]), and competence (*r* = 0.50, *p* < 0.001, 95% CI [0.35–0.62]). In a standard multiple linear regression including all predictors (*R*^2^ = 0.34, adjusted *R*^2^ = 0.29, *F*(9138) = 7.76, *p* < 0.001), empathy (*p* < 0.001), co-operation (*p* = 0.004), and competence (*p* = 0.009) emerged as independent positive predictors of overall impression, whereas the remaining variables were not significant. In contrast, the stepwise regression model, which automatically removed redundant predictors, retained only empathy and co-operation as significant independent predictors (*R*^2^ = 0.34, adjusted *R*^2^ = 0.33, *F*(2142) = 36.9, *p* < 0.001; β = 0.31 and β = 0.40, respectively). This refined model indicates that the effect of competence was largely shared with empathy and collaboration. Together, these results highlight that interpersonal qualities of trainee behaviour—particularly empathy and cooperative communication—are the key determinants of parents’ and patients’ overall perception of care quality within the Kinder-IPSTA program (Table 5).

### 3.4. Qualitative Evaluation of the Questionnaires

A total of 101 of the 149 questionnaires included a free-text comment in response to the question: “When you think about Kinder-IPSTA, what spontaneously comes to mind?”. There were 69 comments from the patients’ parents, 27 from the patients, and 5 on jointly completed questionnaires.

The positive comments related primarily to the communication and care provided by the participants. Twenty comments generally expressed positive views about Kinder-IPSTA. In the other comments, participants were praised for their friendliness (n = 31), good support (n = 12), competence (n = 12), helpfulness (n = 7), interprofessional cooperation (n = 10), and communication (n = 8). Twenty-three comments praised the participants in general for their work. The Kinder-IPSTA concept was mentioned positively in 17 comments. The time dedicated (n = 4) and the continuity of support (n = 2) were also emphasised positively in the comments.

Three critical comments were made, two on parent questionnaires and one on a patient questionnaire. The comments related to the structure of the program (n = 1, “Very good program, a little more structure would give the children more rest.”), the number of people in the room due to the required supervision by the ITW supervisors (n = 1, “Lots of nice, empathetic people. Perhaps sometimes too many for a single child:).”) and the questionnaire (n = 1, “Questionnaire not suitable for children—2 versions necessary”).

## 4. Discussion

In ITWs, members of different professional groups are trained together to enhance future interprofessional collaboration [17,22]. A fundamental prerequisite for the acceptance of this training model by patients, parents, and staff is the assurance of patient safety. In this study, we present our safety concept and assess the satisfaction of patients and their parents with the care provided at the Kinder-IPSTA.

Our safety concept enables us to care for a broad spectrum of patients with congenital heart defects. Notably, patients with severe heart defects or from specific age groups were not excluded from treatment at the Kinder-IPSTA. This ensures that trainees gain experience with a wide range of conditions without compromising patient safety. Compared to the university hospitals and pediatric cardiology departments in Germany participating in the PAN study [21], a larger proportion of severely ill patients were treated at the Kinder-IPSTA. This reflects the generally high proportion of patients with complex congenital heart defects at the Bonn Heart Centre. However, because non-Kinder-IPSTA patients on the ward were not systematically documented, no conclusions can be drawn in this regard.

With its multilevel structure, the safety concept provides a framework that is both easily transferable to other hospitals and adaptable for various specialties. However, the model is labor-intensive, requiring close supervision by medical and nursing ITW supervisors—an aspect that may hinder the implementation of ITWs. In wards with seriously ill children, trainees typically have limited opportunities to work independently [9]. This poses challenges for future recruitment of qualified junior staff in such specialties. At the same time, the need to attract and retain well-trained personnel for specialised wards is becoming increasingly important [23]. Promoting interest and enthusiasm for these fields during training is therefore a logical step [24]. Establishing an ITW enables trainees to assume responsibility for patient care in these areas and to gain valuable experience in pediatric cardiology [18,25].

One drawback of close supervision is that patient admissions and ward rounds may sometimes involve a large number of people in the room. In pediatrics, this can be particularly challenging when caring for anxious children. As one parent noted: “There is only one negative point. Initially, there were too many people at the smear tests and preliminary examinations. That makes it hectic and worsens the situation for the small children.” Similar concerns have been reported in other pediatric studies, in which parents and patients expressed a preference for smaller ward-round teams [26]. According to the ‘hands-off’ principle, the presence of numerous supervisors and trainees should be limited to the initial phase of the Kinder-IPSTA placement, after which trainees gradually assume greater independence. Nonetheless, the supervisory team should remain attentive to this issue and actively minimise the number of people present during examinations and ward rounds whenever possible.

Patient and parent satisfaction at the Kinder-IPSTA was consistently very high across all domains. These findings align with previous evaluations of patient experiences in student training settings [27,28]. In particular, studies on ITWs have demonstrated that the quality of patient care and patient satisfaction are at least equivalent to standard care [6,10,12,25,29]. Similarly, evaluations of monoprofessional pediatric training wards [30] and ITWs in general pediatrics [16] reported high levels of satisfaction among patients and parents. To our knowledge, no previous study has assessed ITWs in a specialised pediatric ward treating seriously ill children. Our study fills this gap and demonstrates that pediatric patients with congenital heart defects can be safely cared for in an ITW setting while maintaining high levels of patient and parent satisfaction.

One explanation for the high satisfaction ratings is the strong commitment and motivation with which trainees care for “their” patients. For the trainees, this work is not part of their everyday routine, but rather an engaging learning process in which they assume responsibility for patients for the first time. Continuity of care is another key factor: the involvement of the same interprofessional team ensures a level of consistency that is otherwise uncommon. As one parent observed: *“It was nice always to have to deal with the same people and not have to change every day.”* This continuity is particularly valuable in pediatrics, where being cared for by familiar individuals helps children reduce anxiety and build trust with their care team. In addition, lunchtime reflection sessions allow trainees to address communication difficulties, crises, and social concerns, fostering constructive solutions and contributing further to patient satisfaction. The comparatively favourable care ratio also enables trainees to spend more time with each patient. As one parent noted: *“Good staffing ratio, high time commitment, good care, much time is taken.”* This combination of dedicated engagement, continuity of care, structured reflection, and time for individualised attention appears to be central to the high levels of satisfaction observed.

Among all questionnaire items, practitioners’ empathy appeared to play a critical role for patients and their parents. In stepwise multiple linear regression analyses, empathy was the only factor with a positive predictive value for overall impression, perceived safety, and likelihood of recommendation. This finding was also strongly reflected in the free-text comments. For example, one patient noted: “They took me seriously when I was scared,” while a parent emphasised: “Super sensitivity, the team showed great empathy for my child and looked after him individually, asked questions about my child before they touched him, great care, we are delighted.”

### Strengths and Limitations

In this study, only patients’ and parents’ perceptions of safety were analysed as an indicator of the safety concept. Although no significant treatment errors or emergency team activations have occurred at the Kinder-IPSTA to date, the systematic assessment of objective patient safety remains an important future focus. A comparative analysis of interprofessional training ward (ITW) and non-ITW patients using a pediatric adaptation of the Global Trigger Tool is currently underway at our institution.

Another limitation concerns the questionnaire used in this study. As no validated instrument for assessing patient and parent satisfaction in pediatric interprofessional training wards currently exists, a study-specific questionnaire was developed and pilot-tested for comprehensibility. While this ensured content validity, formal psychometric validation was not performed. Consequently, direct comparability with studies employing standardized satisfaction measures is limited and should be addressed in future research.

A further potential source of bias arises from the lack of systematic documentation of eligible patients who were omitted. Because patient inclusion was integrated into daily ward operations and guided by safety and educational considerations, maintaining a complete screening log was not feasible. This pragmatic approach reflects real-world clinical conditions but limits precise quantification of potential selection bias.

Moreover, this study was conducted at a single tertiary university hospital with continuous supervision by both a physician and a nurse ITW supervisor, which may restrict the generalizability of the findings to settings with fewer resources. However, ward management has reported that onboarding of former Kinder-IPSTA participants is faster and requires fewer resources, as they are already familiar with workflows and safety routines. Thus, the initially high supervisory effort may be offset by long-term efficiency gains.

Despite these limitations, our study demonstrates for the first time that pediatric cardiology patients can be safely and successfully managed on an ITW, achieving exceptionally high levels of patient and parent satisfaction. We hope that these results will encourage other teaching hospitals to implement similar models and adopt a structured safety concept that enables nursing trainees and final-year medical students to assume greater responsibility in patient care.

## 5. Conclusions

Our experience at the Kinder-IPSTA shows that nursing trainees and final-year medical students safely care for even high-risk pediatric cardiology patients within an ITW, supported by a structured three-level safety concept. Patients and parents reported very high satisfaction across all domains, with empathy, continuity of care, and the trainees’ strong commitment emerging as central factors. Although the lack of a validated questionnaire and the absence of objective patient safety measures such as the Global Trigger Tool represent limitations, our findings provide the first evidence that an ITW model can be successfully implemented in a specialised pediatric ward without compromising safety. By offering trainees responsibility in a closely supervised environment, the Kinder-IPSTA not only ensures high-quality patient care but also fosters enthusiasm for specialised fields, contributing to the recruitment and development of future healthcare professionals.

## Figures and Tables

**Table 1:** Patient characteristics.

Total number of Patients	183
Patients with complete data	170
Female patients: n (%)	81 (47.6%)
Age Mean in years (SD+/−)	7.25 y (+/−5.5)
Range	0–31 y
Length of stay in days Mean (SD+/−)	3 d
Range	1–35 d
Severity of CHD:	
Mild n (%)	52 (30.6%)
Moderate n (%)	28 (16.5%)
Severe n (%)	89 (52.4%)
Missing n (%)	1 (0.6%)

**Table 2:** Frequency distribution of the answers for the various questionnaire items, with the answers of patients and parents cumulated, where 1 was the best response and 4 was the worst. * Patients with complete data.

Item, (n) *	Rating = 1 n (%)	Rating = 2 n (%)	Rating = 3 n (%)	Rating = 4 n (%)
overall impression (n = 149)	121 (81)	28 (19)	-	-
co-operation (n = 148)	121 (82)	26 (17)	1 (1)	-
Informed (n = 149)	110 (74)	37 (25)	2 (1)	-
Advised (n = 148)	116 (78)	30 (21)	2 (1)	-
Competence (n = 147)	107 (73)	39 (26)	-	1 (0,7)
Empathy (n = 149)	132 (88)	16 (11)	1 (1)	-
Encouragement and support (n = 149)	130 (87)	18 (12)	1 (1)	-
Taken seriously (n = 149)	136 (91)	12 (8)	1 (1)	-
Devoted time (n = 147)	128 (87)	18 (12)		1 (1)
Safety (n = 149)	115 (77)	32 (21)	1 (1)	1 (1)

**Table 3:** Comparison of the frequency distributions of the answer to the question: “How safe did you feel when you were cared for by the Kinder-IPSTA team” by patients and parents.

	Parent Questionnaires (n = 103)	Patient Questionnaires (n = 33)
“Very safe” n (%)	75 (73)	29 (88)
“Safe” n (%)	27 (26)	4 (12)
“Unsafe” n (%)	0	0
“Very unsafe” n (%)	1 (1)	0

**Table 4:** Comparison of the frequency distributions of the answer to the question: “How safe did you feel when you were cared for by the Kinder-IPSTA team” of patients with different degrees of severity of CHD. Answers from patients and parents were accumulated.

	Mild CHD (n = 45)	Moderate CHD (n = 27)	Severe CHD (n = 64)
Very safe (1) n (%)	32 (71)	23 (85)	50 (78)
Safe (2) n (%)	13 (29)	4 (15)	13 (20)
Unsafe (3) n (%)	0	0	0
Very unsafe (4) n (%)	0	0	1 (2)

**Table 5:** Multiple linear regression models for predictors of perceived safety and overall impression. β = standardized coefficient; CI = confidence interval; R^2^ (adj.) = R-squared (adjusted).

Dependent Variable	Predictor	β	95% CI	*p*	R^2^ (adj.)
Perceived Safety	Competence	0.31	[0.17–0.44]	<0.001	0.56 (0.54)
	Empathy	0.30	[0.25–0.63]	<0.001	
	Advice about treatment	0.28	[0.16–0.45]	<0.001	
	Time devoted	0.29	[0.15–0.54]	<0.001	
	Taken seriously	−0.20	[−0.58–−0.07]	0.014	
Overall Impression	Co-operation	0.40	[0.24–0.51]	<0.001	0.34 (0.33)
	Empathy	0.31	[0.18–0.51]	<0.001	

## Data Availability

The data presented in this study are available on request from the corresponding author due to privacy reasons.

## Abbreviations

The following abbreviations are used in this manuscript:

ITW	Interprofessional Training Ward
CHD	Congenital Heart Defects
WHO	World Health Organization
IPSTA	German Abbreviation—Interprofessionelle Ausbildungsstation, meaning: Interprofessional Training Ward
CRM	Crisis Resource Management
PALS	Pediatric Advanced Life Support
PBLS	Pediatric Basic Life Support
ISBAR-R	Identification, Situation, Background, Assessment, Recommendation-Read back
MED	Medical Emergency Team
SPSS	Statistical Package for the Social Sciences
